# A Proposed System for Temperature Measurement During Tensile Testing

**DOI:** 10.3390/s25175494

**Published:** 2025-09-04

**Authors:** Marius Andrei Mihalache, Vasile Merticaru, Vasile Ermolai, Liviu Andrusca, Nicanor Cimpoesu, Florin Negoescu

**Affiliations:** 1 Department of Machine Manufacturing Technology, Gheorghe Asachi Technical University of Iasi, 700050 Iasi, Romania; marius-andrei.mihalache@academic.tuiasi.ro (M.A.M.); vasile.ermolai@academic.tuiasi.ro (V.E.); florin.negoescu@academic.tuiasi.ro (F.N.); 2 Department of Mechanical Engineering, Mechatronics and Robotics, Gheorghe Asachi Technical University of Iasi, 700050 Iasi, Romania; liviu.andrusca@academic.tuiasi.ro; 3 Department of Materials Science and Engineering, Gheorghe Asachi Technical University of Iasi, 700050 Iasi, Romania; nicanor.cimpoesu@academic.tuiasi.ro

**Keywords:** thermal imaging, 3D printing, tensile testing, ANOVA, FEM, SEM

## Abstract

Integration of thermographic imaging with in situ scanning electron microscopy (SEM) analysis may aid in quantifying thermal–mechanical behavior during tensile testing of 3D-printed polymers, which gives information about fracture mechanics, including the associated thermal phenomena. Upon fracture, samples exhibit changes in the thermal field, which is interesting because temperature fluctuations can affect material integrity. The paper introduces printing parameters to demonstrate a thermal measurement system’s sensitivity in detecting variations in mechanical response due to controlled changes in the process. Employing scientific methods, one can extrapolate results to a wider class of materials such as thermoplastics. Analysis of variance (ANOVA) is key in the design of experiments (DOE) if one wants to analyze the effect of factors and interactions. It has been used with the purpose of reducing the risk of type I errors (i.e., false positives). The finite element method (FEM) highlights temperature distribution in the area of interest and confirms recorded data. The particularly developed research experiments are carried out in a laboratory environment. Different samples are subjected to tensile tests under the evaluation of changes in the thermal field. SEM analysis is also widely used in fracture analysis to understand failure modes (ductile vs. brittle, crazing, delamination, and others). Thus, the paper aims to present a custom setup comprised a thermal camera pointed at samples during tensile testing that would serve as a reliable assessment system that accounts for the substitution of a sensor-based environment but is still fully capable of validating the measurement approach.

## 1. Introduction

The combination of thermal imaging and in situ observation, aligned with mechanical testing, offers a coherent measurement system for comprehensive material evaluation. It is generally accepted that printing parameters have a great influence on the final part’s mechanical integrity and toughness. Hamidi et al. have studied the influence of printing parameters of thermoplastic polymers. By blending two filaments they were able to detect how various ratios impact final products by means of differential scanning calorimetry (DSC) and thermogravimetric analysis (TGA). Not only printing procedures are responsible, but also different blends that, in their case, accounted for a reduced cold crystallization temperature among others, which gave 64% reduced strength but better ductility. By using different strain rates for tensile tests, the authors have found a relation to the rate-dependent properties of the PLA/TPU polymer [1].

Jin et al. have studied the thermal properties of some 3D-printed monofilaments. At a certain value of the monofilament diameter, they have found that the tensile strength reaches a maximum of 48.3 MPa in the case of carbon fiber-reinforced polylactic acid (CF-PLA) and as CF amount is increased up to 8 vol%, a high thermal stability is achieved. A single PLA-based board shows numerous fusion interfaces, which may account for a tensile strength of just 22.1 MPa, which is half the strength of CF-based ones. Thermogravimetry revealed that the thermal conductivity of the CF-based monofilament is higher than the average PLA one. However, if the threshold of 8 vol% is exceeded, thermal stability is affected by favoring the appearance of more cracks [2].

Švantner et al. tackled the problem of thermographic devices that exhibit limited accuracy, often rendering them inadequate for certain applications. They propose a built-in active reference element that leads to enhanced measurements, thus obtaining pertinent results in a given range. It shows the potential for commercial devices to be used in precision-based applications by successfully substituting more expensive equipment [3].

Researchers have strived to find ways for suitable thermal management. Analyses of thermal performance were carried out by Pei et al. on 3D-printed polymer vacuum insulation panels known for their low thermal conductivity. Their thermal insulation performance derives directly from thermal conductivity, as it was found that it is very sensitive to the emissivity of the solid material when the pressure levels are low. However, PLA for example, exhibits higher emissivity in the infrared region, which explains the behavior of samples under temperature fluctuations. There is a reason why researchers are aiming for lower values for thermal conductivity, as it opens the path for other methods for increasing strength of printed parts [4].

Researchers have addressed different types of tests to assess the strength of materials, among which are tensile ones under thermal cycling conditions. By varying temperature conditions, Kang et al. were able to correlate material strength with temperature variations. They have used the Matsuoka model and achieved up to 90% more strength for their polymethyl methacrylate (PMMA) samples because the polymers react upon heating beyond the glass transition temperature (Tg) but were able to maintain a consistent elastic modulus for the studied samples batch [5].

By deploying thermal data augmentation techniques, Khoshkbary-Rezayiye et al. have managed to combat limited datasets. Their approach, which uses deep learning, aims at the automation of defect detection processes. By generating synthetic sub-surface defects by means of finite element modelling (FEM), one could obtain synthetic thermal images, that when overlapped onto reals ones, may be used for training the learning algorithm. This method shows the potential of thermal imaging in the detection of defects in pipes but may be extended to others as well [6].

Other researchers have used scientific methods like ANOVA, Taguchi, and grey relational analysis (GRA) to find ways to improve the mechanical performance of 3D-printed samples through thermal annealing. These methods also produce optimal results in in form of tensile strength, flexural strength, compressive strength, and impact resistance. Kahya et al. found a significant effect of annealing temperature and time on the tensile properties of their PLA-based samples which exhibited approximately a 25% increase in strength after 200 min of annealing at 90 °C, thus facilitating beneficial phase transition. ANOVA revealed that temperature was the primary factor, accounting for 94.46% of the variance almost independent of time. By employing differential scanning calorimetry (DSC) results, it was confirmed that the process of annealing enhances and promotes molecular alignment, which gives more stable crystalline regions. This type of research may be very well continued by assessing the influence of temperature fluctuations in 3D-printed models [7].

Fujita et al. are evaluating fatigue damage of carbon-fiber-reinforced plastic (CFRP) structures by taking into account thermal diffusivity and specific heat capacity retrieved by lock-in thermography. After numerous fatigue cycles, which produced microstructures changes and degradation, values of thermal diffusivity decreased and triggered an increased volumetric heat capacity under fatigue loading [8]. Thus, thermal evolution may be captured and assessed as one of the main output factors of certain mechanical processes. Demarbaix et al. have also studied defect detection by using active thermography. They have established two benchmarks that set a detectability threshold and look for defects as a result of 3D printing. Successful detection is achieved through reflection and transmission modes as reflection avoidance is not necessary. It shows the potential of using thermal equipment for early detection [9].

Roudný et al. have reviewed FDM-ready composites that are thermal conductive (TC). It is pointed out that, regularly, anisotropy may be present as TC being higher in the print direction. Even though manageable by 3D printing, oriented fibers are not preserved after process completion due to their inability to maintain molecular orientation after filament reheat, which often occurs when the polymer that is molten stretches under stress-induced conditions to form macromolecules. It has been found that it is possible to achieve a certain TC in a given time interval that is comparable to drawn fibers’ molecular orientation by 3D printing at high speeds. Of course, the addition of conductive fillers helps in the sense that smaller particles form a denser network with improved TC. One of the solutions is to build a segregated structure by using coextrusion that would allow a shell cover to be created. However, there are limitations, as adhesion between the network structure and polymer matrix must be improved [10].

Interlayer adhesion was also studied by Nguyen et al., which revealed that it also depends on polymeric chain mobility because as soon as thermal energy accumulates that will determine when layers fuse and bond. Another interesting approach focuses on the post-processing of parts obtained by means of 3D printing by introducing an additional thermal treatment that further strengthens their mechanical toughness through interlayer interaction. Others have built custom chambers that allowed envelope temperatures to be controlled, which led to enhanced neck growth and improved strength of the interlayers. By employing a time–temperature superposition (TTS), they have investigated the creep modulus of their samples, which exhibited a greater value than that of the untreated specimens that might be explained by good local relaxation and greater polymer chain flexibility. That translates into less visible interfacial voids and porosity on treated specimens and also impacts tensile strength [11].

Castillo et al. tackled the relationship between temperature profiles and ultimate tensile strength (UTS) by using a cyber–physical production system (CPPS) that analyzed certain thermal parameters, such as layer thickness, printing speed, extrusion temperature, and bed temperature. The first one was found to be the most influential as UTS registered a 17% increase. The study revealed that good mechanical strength is possible through optimal thermal transition between deposition, adhesion, and layer solidification [12].

The present study aims to present a functional setup that may substitute dedicated, highly expensive, sensor-based systems for the assessment of thermal evolution of 3D-printed samples that were subjected to tensile tests. Video files were recorded for each sample in order to capture peak values and temperature evolution, in time, in such a manner that it would encompass a monitoring system. Statistical methods were employed that highlight tendencies, depending on input parameters. Scanning electron microscopy (SEM) was used, as well as numerical simulations based on finite element analyses (FEA), that revealed stress distribution as tensile testing was carried out. The proposed setup, which includes the thermal imaging equipment, may account for a sensor-based system used for monitoring anisotropy and mechanical strength in FDM printing systems and is susceptible to system capability analysis. The results confirm initial assumptions and allow statistical analysis with valuable insights. Improvements in terms of printing parameters may overcome defects revealed by fracture analysis. Limitations are present due to equipment capabilities. The authors acknowledge that there may be a research gap because by studying similar work we aim to demonstrate the diversity of scientific approaches and experimental frameworks that address similar concerns in the field while using a custom evaluation system.

## 2. Materials and Methods

**Figure 1:**
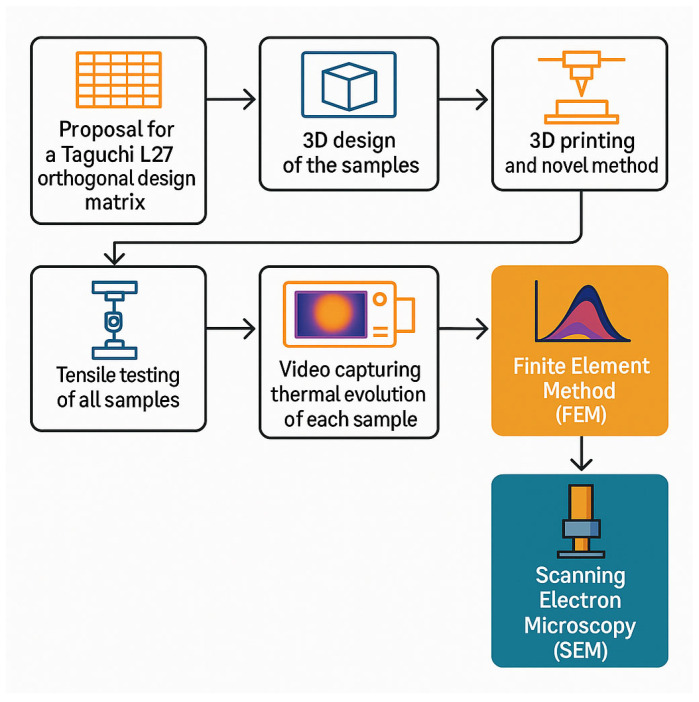
Workflow schematics of experimental testing setups and methods.

The methodological framework is designed to be replicable by researchers in related fields of mechanical engineering and others, offering a structured approach that integrates both the experimental setup and several analyses that are used as part of the evaluation system as a whole. By documenting these stages, the paper aims to prove reproducibility and transparency, enabling others to adopt or adapt the system according to their specific investigative needs. The proposed workflow can be observed in Figure 1.

### 2.1. Experimental Testing Setup

The proposed setup involves the HTI HT-18 thermal imaging camera, manufactured by Dongguan Xintai Instrument Co., Ltd. (Dongguan, China), the People’s Republic of China, mounted on a tripod. It has a vanadium oxide uncooled infrared focal plane detector with a resolution of 256 × 192 and a cell size of 12 μm. Measurement accuracy varies from −15 °C to 550 °C with ±2% error and a temperature measurement resolution of 0.1 °C. Calibration involved adjusting the emissivity value to match the object being measured, thus for matte surfaces a default of 0.95 was used.

**Figure 2:**
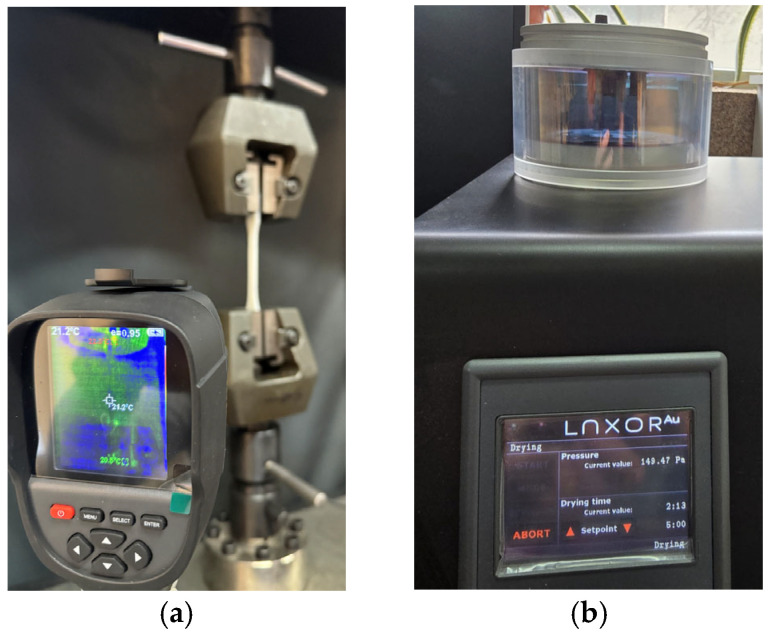
Setup of different stages: (**a**) image taken in the laboratory premises for tensile testing and (**b**) image taken in the laboratory premises for scanning electron microscopy.

The equipment was placed in front of the universal mechanical testing machine, WDW 50, manufactured by Jinan Hensgrand Instrument Co., Ltd. (Jinan, China), the People’s Republic of China (see Figure 2a), used for tensile testing of specimens and structures of various shapes, including cables, profiled bars, plates, springs, and others. The machine is driven by an AD800 closed-loop automated measurement and control system. The driving software (i.e., WinWdw Electronic Universal Testing Machine Measure and Control System, version 2.15.P.E) automatically calculates and stores tensile experimental data such as strength, yield strength, modulus of elasticity, or elongation at break. It has a 50 kN load cell with a 600 × 600 mm^2^ testing area. The measurement scale ranges from 0.4% up to 100% full scale (FS). Speed control allows increments of 0.02–100% (FS/min), with the equipment being able to move at rates of 0.005~500 (mm/min).

It used the ISO 527-2:2012 type 1B standard for sample preparation [13]. The 3D models were designed in 3D CAD type of software (i.e., Solid Edge 2023, teacher licensed, version 2210). Scanning electron microscopy was carried out using a SEM VegaTescan LMH II, manufactured by Tescan Orsay Holding, Brno, Czech Republic (see Figure 2b).

The aim of this study was to use additive manufacturing to obtain 3D-printed samples that will be subjected to tensile testing. The process requires layer-by-layer deposition to build a part directly from a digital model, with minimal material waste. The fabrication method is favored because several process-related factors exhibit great influence. Material selection affects strength, ductility, and thermal resistance. Poor interlayer bonding leads to weaker mechanical strength as print orientation and path significantly affect anisotropic properties. Process parameters could affect porosity, warping, residual stresses, and surface finish, and uneven cooling can cause internal stresses or cracks.

The authors have tested multiple infill patterns for the 3D-printed specimens with their aim to achieve ones that would exhibit enough temperature variation to be captured by the thermal imagining equipment. It was found that neither produced satisfactory results as fracture occurred too rapidly without any significant burst of thermal behavior. Thus, the present study proposes a printing strategy that would compensate for that.

**Figure 3:**
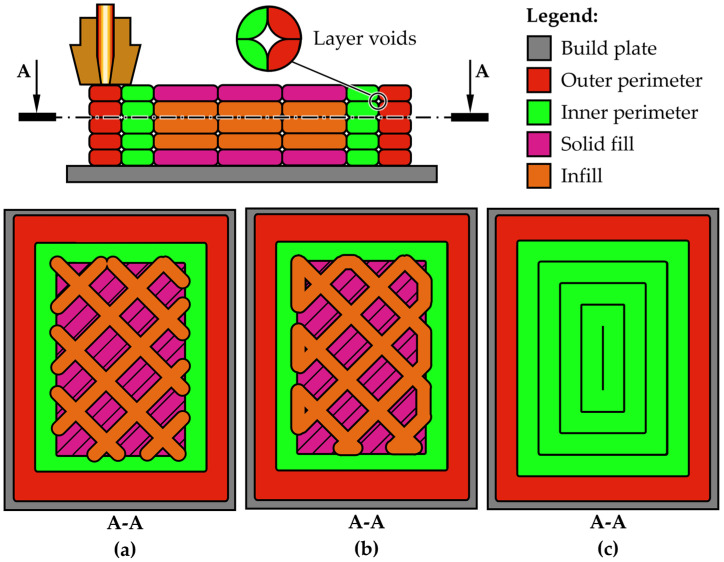
Snapshot from the slicer with the proposed printing strategy: (**a**) standard infill; (**b**) infill with connecting lines; and (**c**) infill with walls.

Specimens were 3D-printed on a Prusa Mk3s+ type of 3D printing equipment and the slicer used was an Orca slicer 2.3.0-beta, which is an open-source type of software. The above-mentioned strategy involves printing concentric walls as opposed to an infill pattern, as observed in Figure 3. The grip area was extended from 17.5 mm to 30 mm for a better grip, as it was found that the proposed printing strategy allows fractures only in the calibrated area of the specimen since each one exhibited the same behavior during testing.

This type of printing creates layer voids that behave differently depending on the type of material chosen and does account for the brittleness or ductility of the test specimens. It is expected that fibers upon fracture should elongate or even curve as the specimen turns ductile in opposition to brittle ones that should exhibit a sudden, clean cut.

The first type of infill shows the way the slicer commands the 3D printer to layout each layer. One can see that when the infill pattern meets the inner walls depicted in green, there is a piercing by a tiny fraction, thus creating weak points that will act as stress concentrators (see Figure 3a). This is the reason why the samples prematurely fail. The slicer shows that it enters the wall structure with about 0.1 to 0.2 mm out of the wall’s 0.4 m thickness, to allow a steady fusion. The second graphical representation proposes that the infill would take advantage of connecting lines instead of points of intersection (see Figure 3b). This allows a diminishment of the stress concentrators effect but still fails to achieve this study goal of capturing thermal burst upon fracture. The third strategy does not use infill but favors concentrically printing walls with offset (see Figure 3c). This produces layer voids when viewed in section as scanning electron microscopy will reveal. The approximate shape is presented in detail in Figure 3. This strategy offers the benefits of improved toughness along the fiber, because the printed sample is essentially a package of very long, stacked filament.

The design of experiments (DOE) followed a Taguchi L27 orthogonal array, incorporating five input factors, each at three levels of variation. The selected parameters for the 3D-printed samples included: material type, raster height, raster width, material flow rate, and pulling speed during tensile testing, as detailed in Table 1.

The study utilizes three different materials: a white acrylonitrile butadiene styrene (ABS) filament from the company BASF, Emmen, Germany, a red acrylonitrile styrene acrylate (ASA) filament from the company FormFutura, Amsterdam, The Netherlands, and a black recycled polyethylene terephthalate glycol-modified (rPETG) filament also from FormFutura, Tarweweg, the Netherlands. Before printing, all filaments were dried at 70 °C for six hours. During the printing process, the filaments remained in a silica-conditioned enclosure to prevent moisture absorption. The printing parameters adhered strictly to the manufacturers’ specifications. For the 3D printer parameters, the raster height varied at 0.15 mm, 0.25 mm, and 0.35 mm, while the raster width levels were set at 0.4 mm, 0.6 mm, and 0.7 mm. These parameters controlled the cross-sectional dimensions of the extruded filaments [14]. The material flow rate was adjusted to 100%, 105%, and 110% of the nominal volumetric flow rate, based on the hypothesis that increased flow rates would reduce internal voids between filament layers. Specimens followed a modified version of the ISO 527-2:2012 type 1B standard, incorporating a 30 mm grip zone. For each parameter combination, five dog bone-shaped specimens were printed using a Prusa Mk3s+ enclosed 3D printer (Prague, Czech Republic), equipped with a 0.6 mm nozzle. After conditioning for 24 h in a controlled laboratory environment (20 °C and 50% relative humidity), the specimens were subjected to tensile testing at three pulling speeds: 50 mm/min, 100 mm/min, and 200 mm/min. It was found that at those values there are results that can be captured by the thermal camera. The mechanical tests were carried out on the WDW 50 [15]. The three above-mentioned types of filaments were chosen among others for their intrinsic thermal properties such as heat capacity (*Cp*), thermal conductivity (*k*), and thermal diffusivity $\left(\alpha \;=\;k/\rho \cdot Cp\right)$, where (*ρ*) stands for density. Approximate values are presented in Table 2.

Each of these polymers exhibits distinct thermal behavior. The properties of ABS, an amorphous thermoplastic, offer balanced thermal insulation and dimensional stability during printing [16,17]. The attributes of ASA not only allow for similar processability as ABS but also impart improved resistance to weathering and UV degradation, thus extending its use to outdoor applications where thermal and environmental stability are required [17,18]. On the other hand, recycled PET, a semi-crystalline thermoplastic, exhibits a more thermally complex behavior. Due to post-processing and thermal history, rPETG demonstrates a wide variability in its crystallinity (20–35%), which influences its thermal response during manufacturing and end-use [16,18,19].

### 2.2. Setup for Thermal Variation Assessment of the Tensile Test Using Finite Element Method (FEM)

The use of the finite element method (FEM) was aimed at replicating experimental tests when fracture of a test specimen occurs. For that purpose, an Ansys researcher license was used. By simulating the entire experimental test, one could obtain similar results by means of finite element analyses (FEA) that would thus be validated. Furthermore, stress and strain distributions may be revealed so that conclusions may be formulated regarding the thermal behavior of specimens under given conditions that otherwise would be difficult to assess.

**Figure 4:**
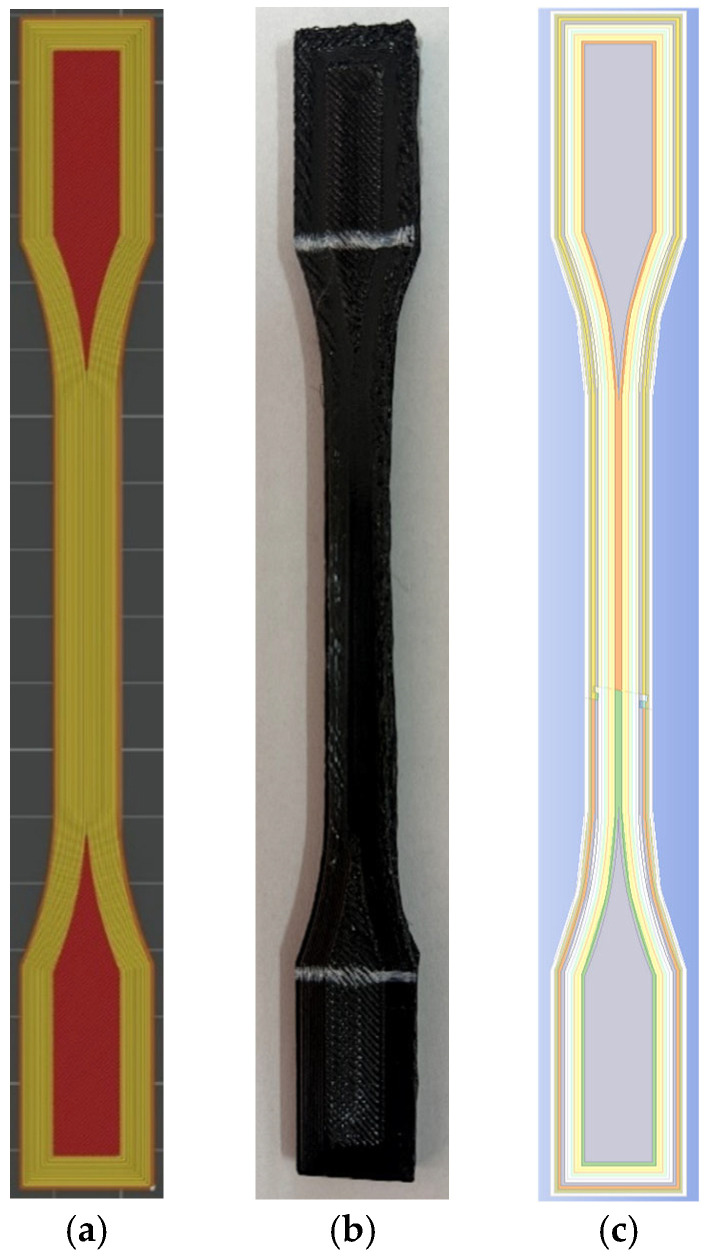
Specimen model: (**a**) plot of 3D printing process manipulation; (**b**) 3D intermediate printed sample made of ABS; and (**c**) 3D CAD model replica of the wall infill pattern.

The present study uses samples obtained by a 3D printing process manipulation in the form of a multiple concentric walls internal layout that favors increased toughness of each specimen inside the calibrated zone during the tensile tests (see Figure 4a).

The 3D model was imported to the Ansys Explicit Dynamics module in Parasolid format. The reason that this module was first used is that FEM aims to replicate one tensile test, including thermal assessment. This is made possible by importing the results of the above-mentioned module into another one, namely Steady-State Thermal module.

It was chosen to use ABS from the software library, which was assigned a non-linear behavior. The material exhibits isotropy, thus transforming the analysis into an ideal setup. An anisotropic type of ABS would predict failure along weaker planes, such as inter-layer boundaries in fused deposition modelling (FDM) printed parts. This makes results prone to overestimate ductility. To overcome this, the solution was to implement a direction-dependent material model in FEM in the form of an orthotropic material definition, which is based on experimental stress–strain data in different orientations retrieved from previous research [20]. Future endeavors will also use anisotropic failure criteria like Tsai-Wu, Hashin, or maximum stress theory in FEM to capture directional weaknesses.

The connection branch uses no contacts or body interactions since the 3D model is interpreted as a single body.

Mesh wise, there were several software tools used.

It was assumed that failure would occur in the calibrated area because boundary conditions were set in such a manner that would favor it. Experimental tests revealed that the fracture area is slightly elongated and very abruptly shaped, with leftovers that are irregular and randomly distributed, leading to the conclusion that the triangle type of finite elements must be used for the simulation to succeed.

**Figure 5:**
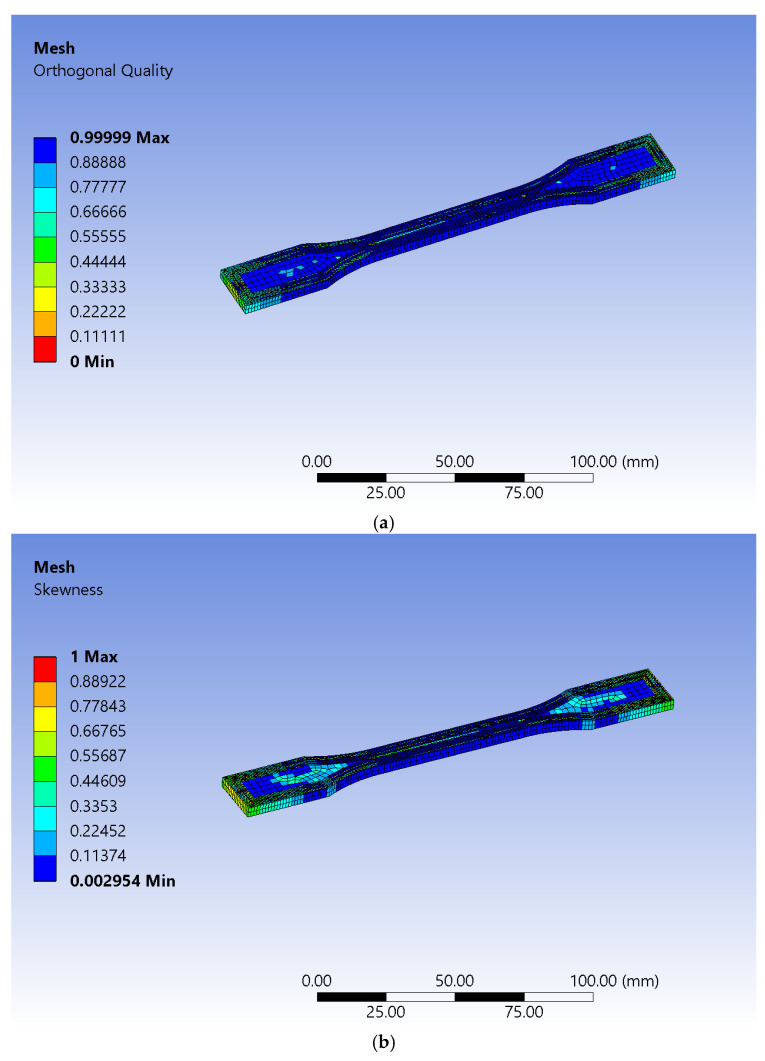
Mesh quality assessment: (**a**) orthogonal and (**b**) skewness.

The body sizing method, in combination with patch conforming that uses both quadratic and triangle-shaped elements, was first imposed. It led to inaccurate results since it was obvious that there was a need for higher-order types of finite elements. Thus, the Hex-dominant method that forces all quads was used. That produced hexagonal elements with eight nodes (Hex8) and wedge-shaped ones with six nodes (Wed6), with acceptable orthogonal quality in the calibrated zone, as can be observed in Figure 5a. Skewness assessment showed that the areas affected are outside the calibrated zone, as can be seen in Figure 5b. The process of meshing produced 19,482 nodes and 6676 elements.

**Figure 6:**
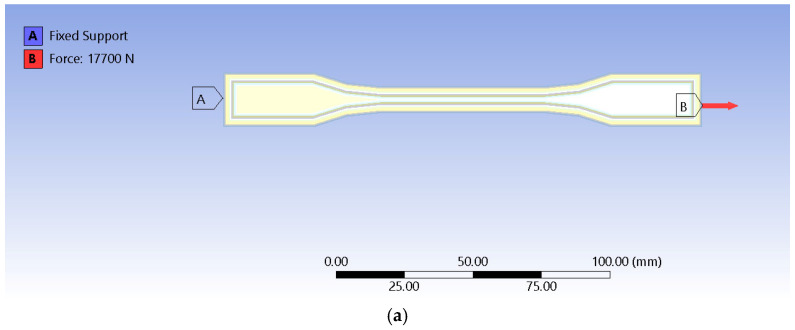

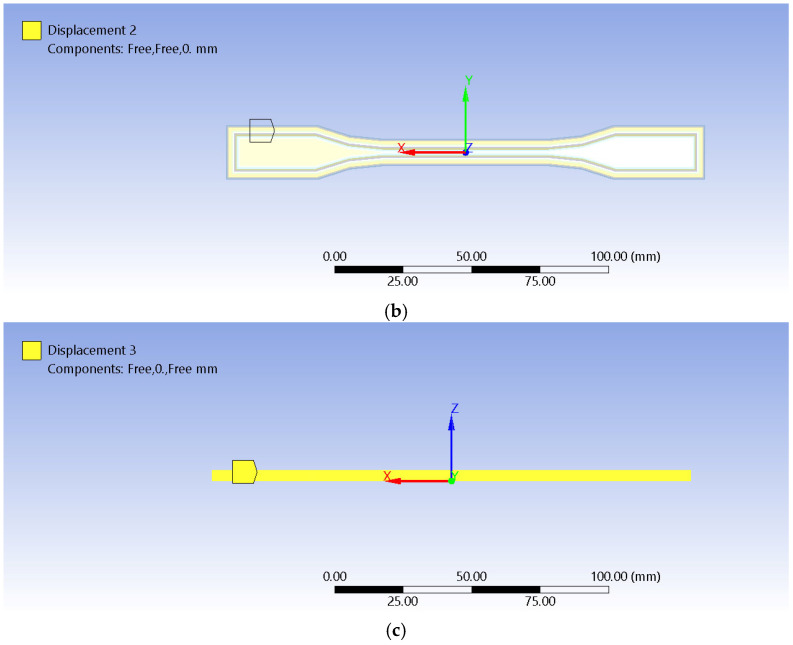
Boundary conditions: (**a**) force type-of-load and fixed support applied at both ends; (**b**) zero displacement on the *Z* axis for upper and bottom faces; and (**c**) zero displacement for lateral faces.

Boundary conditions include a force type-of-load that received the same value as one that has been recorded in experimental testing and a fixed support that holds the sample in place. Thus, tensile testing conditions are met. However, because of the skewness that the mesh quality inquiry has revealed, additional conditions were imposed. Displacements of zero value on the *Z* axis were introduced for the top and bottom faces, as well as on the *Y* axis, for the lateral faces of the model, as can be observed in Figure 6.

Analysis settings had no automatic mass scaling and a limit of one for the geometric strain limit, which corresponds to 100%. Because FEM aimed to achieve readings as accurately as possible regarding thermal variation, all experimental tensile tests were performed in a laboratory room with temperatures that varied from 19.6 °C to 20.4 °C. Thus, for the randomly chosen test for replication, the temperature reading was of 20.1 °C, which was inputted as an initial condition.

FEM is considered valuable because it allows the assessment of potential failure points that might not be captured in traditional statistical analysis by the identification of stress concentrations. Enhancing statistical models with physical insight often relies on statistical variation of outputs (e.g., stress, displacement). In this regard, FEM allows for the simulation of a wide range of input parameter variations and the incorporation of physics-based constraints into statistical analysis, thus making it more robust.

### 2.3. Setup for Scanning Electron Microscopy (SEM)

The 3D-printed samples after breakage were analyzed microscopically and macroscopically using a stereomicroscope and a scanning electron microscope (VegaTescan-LMHII, SEM, VegaScan, Brno-Kohoutovice, Czech Republic, SE detector, 10 kV gun power supply) with the software solution VegaTC version 3.5.0.0.

**Figure 7:**
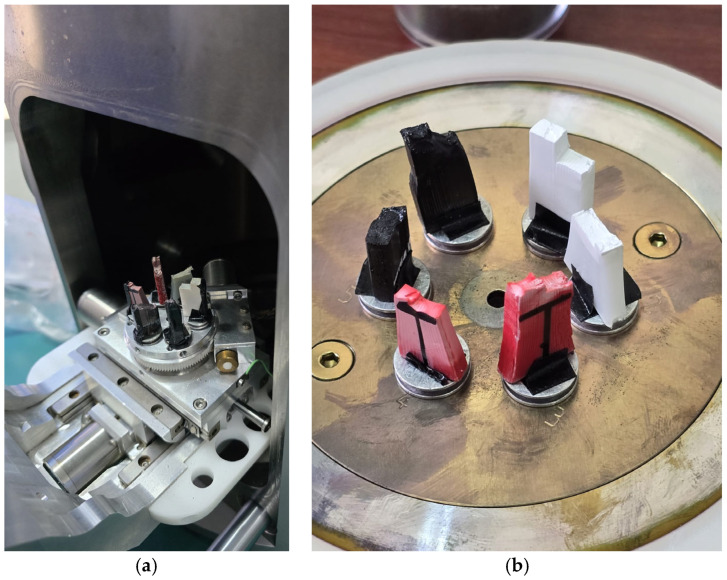
Preliminary steps for SEM analyses: (**a**) view of setup and (**b**) sample fixing procedure.

For scanning electron microscopy, the samples were coated with a 7 nm gold layer using Luxor Au plasma deposition equipment produced by Luxor Tech, Nazareth, Belgium, after preliminary vacuuming of the enclosure. Images of the setup from the laboratory are presented in Figure 7.

The analysis followed a standardized protocol: images were converted to 8-bit grayscale, the scale was set based on the magnification (0.2 mm or 0.02 mm), and regions of interest were cropped to sizes of 0.12 × 0.12 mm^2^ and 1.25 × 1.25 mm^2^, respectively. Thresholding was applied using the Otsu method, followed by particle analysis to quantify surface porosity. Higher magnification images (500× and 1000×) were used to evaluate micro-void distribution, while lower magnification images (typically 100×) were employed to estimate the percentage of surface area occupied by interlayer voids.

## 3. Results

**Figure 8:**
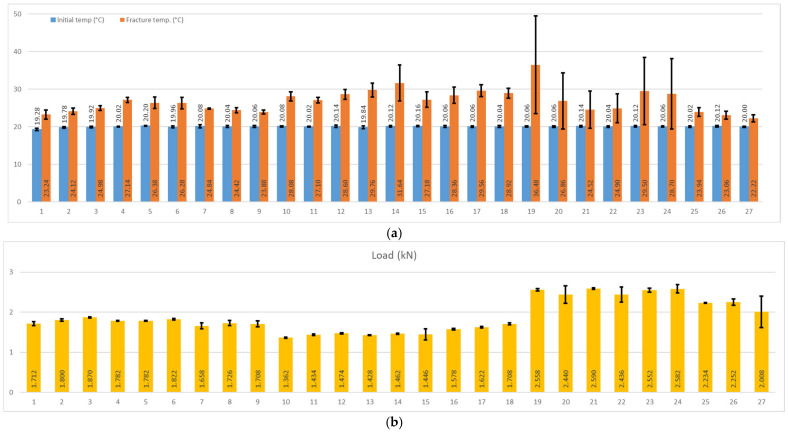

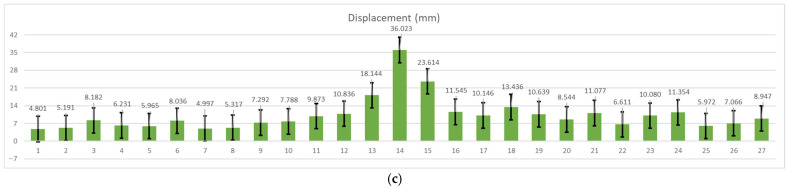
Plots: (**a**) temperature variation; (**b**) load; and (**c**) displacement.

Results from tensile tests were recorded and used for a clear view of each type of filament used, as can be observed in Figure 8, as plots were designed for all 27 samples.

### 3.1. Statistical Analysis Results

**Figure 9:**
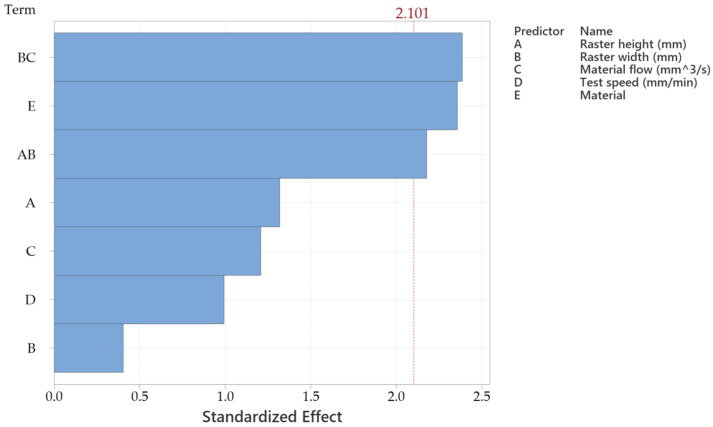
Pareto chart of the standardized variable effects for the samples’ temperature at fracture.

Data evaluation was performed using Minitab 20 software. Analysis of variance (ANOVA) was conducted at a significance level of α = 0.05. To enable comparison across different units and variable types, all variables were standardized by subtracting the mean and dividing by the standard deviation. The Taguchi L27 orthogonal design was used because the study aims at statistical relevance, which can be achieved by a wider array of tested specimens. Beginning with the fracture temperature response, the Pareto chart from Figure 9 indicates that material type exerts the most significant influence [21]. The material flow directly influences the raster width and, consequently, the contact area at the interface level in the way that a slight increase results in an additional volume of molten material, which tends to fill the voids between adjacent rasters. This surplus partially overlaps the previously deposited ones, thereby enhancing interlayer adhesion and contributing to increased tensile yield and strength. It is highlighted that the interaction between raster height and raster width plays a critical role in determining the tensile properties, as it affects the cross-sectional geometry of the rasters.

As shown in Table 3, the material parameter achieved an F-value of 4.29 and a *p*-value of 0.03. Although the other main factors also affected the temperature response, their individual effects were less significant than certain interaction effects. In particular, the raster width × material flow and raster height × raster width interactions demonstrated statistically significant contributions to fracture temperature variation [22].

**Figure 10:**
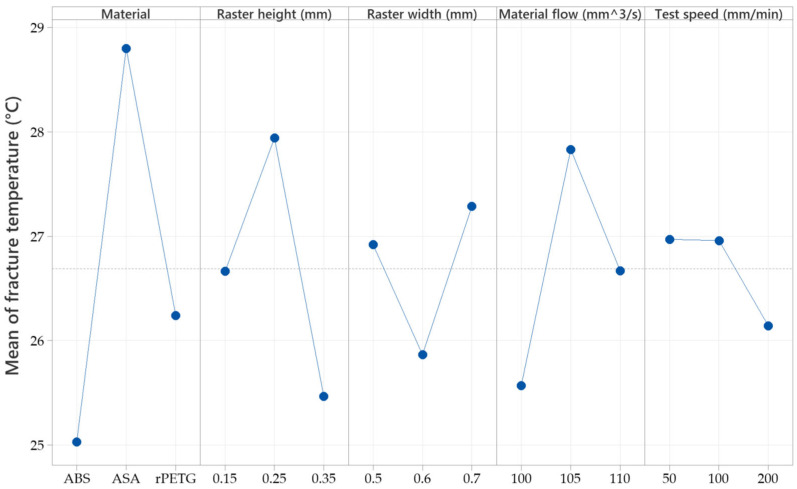
Factorial plots for the samples’ temperature at fracture.

The factorial plots in Figure 10 reveal that the highest fracture temperature occurred in ASA samples printed with a raster height of 0.25 mm, a raster width of 0.7 mm, a material flow of 105%, and pulled at a speed between 50 and 100 mm/min.

**Figure 11:**
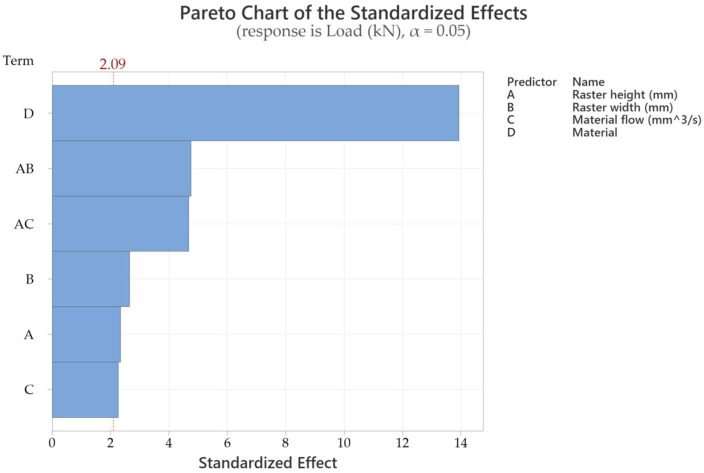
Pareto chart of the variable standardized effects for the tensile load.

Regarding the load capacity response, the Pareto chart from Figure 11 confirms that material type again plays a dominant role, with an F-value of 334.31 and a *p*-value below 0.001.

In this case, additional main effects also contributed significantly, specifically raster width, raster height, and material flow, in descending order of influence [23]. Pulling speed did not show a statistically significant effect. Furthermore, the raster height × raster width and raster height × material flow interactions significantly influenced the load-bearing capacity, as shown in Table 4.

**Table 4:** Analysis of variance of the samples’ tensile load (kN) at peak.

Source	DF	Seq SS	Contribution	Adj SS	Adj MS	F-Value	*p*-Value
Regression	7	4.24848	97.55%	4.24848	0.60693	108.19	0.000
Raster height (mm)	1	0.03092	0.71%	0.03092	0.03092	5.51	0.030
Raster width (mm)	1	0.03957	0.91%	0.03957	0.03957	7.05	0.016
Material flow (%)	1	0.02880	0.66%	0.02880	0.02880	5.13	0.035
Material	2	3.89919	89.53%	3.75072	1.87536	334.31	0.000
Raster height × Raster width	1	0.12712	2.92%	0.12712	0.12712	22.66	0.000
Raster height × Material flow	1	0.12288	2.82%	0.12288	0.12288	21.91	0.000
Error	19	0.10658	2.45%	0.10658	0.00561		
Lack-of-Fit	1	0.01338	0.31%	0.01338	0.01338	2.58	0.125
Pure Error	18	0.09320	2.14%	0.09320	0.00518		
Total	26	4.35506	100.00%				

**Figure 12:**
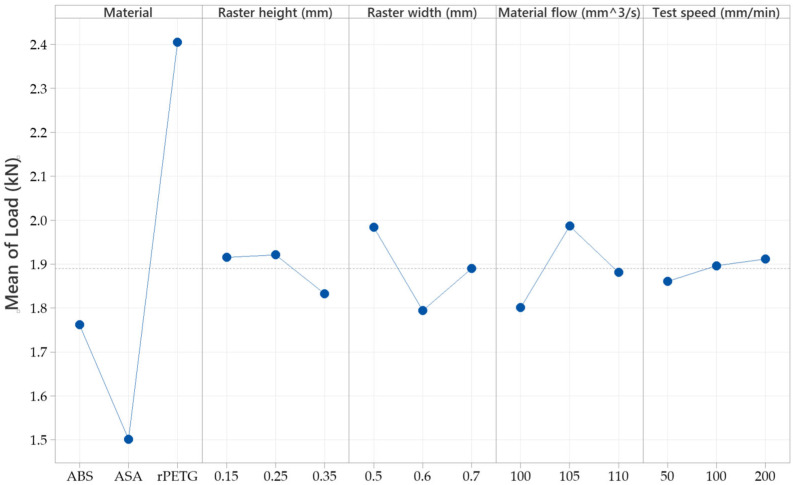
Factorial plots of the samples’ tensile load at peak.

The factorial plots in Figure 12 indicate that the highest load capacity was achieved in rPETG samples printed with a raster height of 0.25 mm, a raster width of 0.5 mm, and a material flow rate of 105%.

### 3.2. Finite Element Analysis (FEM) Results

Belgen et al. have pioneered measurements of thermal infrared radiation as a valuable tool in the assessment of mechanical properties through their stress pattern analysis by measurement of thermal emission (STATE) proposal [24]. Others have followed and have established a ground basis for future research [25,26].

Results were requested as equivalent stress evaluated under von Mises criteria. We know that the force *F* measured in a uniaxial tensile test is used to compute the nominal stress *σ* by the following:

(1) $$\sigma =\frac{F}{A_{0}}$$

where *A*_0_ is the cross-sectional area of the specimen.

Under uniaxial loading, the von Mises stress *σ_v_* is given by the following:

(2) $$\sigma_{v}=\sigma$$

However, in a general 3D stress state, the von Mises stress is calculated as follows:

(3) $$\sigma_{v}=\sqrt{\frac{1}{2}\left[{\left(\sigma_{1}-\sigma_{2}\right)}^{2}+{\left(\sigma_{2}-\sigma_{3}\right)}^{2}+{\left(\sigma_{3}-\sigma_{1}\right)}^{2}\right]}$$

For a uniaxial tensile test, the principal stresses are the following:

σ_1_ = σ, which is applied stress in the tensile direction;

σ_2_ = 0, assuming no lateral stress in transverse directions;

σ_3_ = 0, assuming no lateral stress in the thickness direction.

Substituting these into the von Mises equation,

(4) $$\sigma_{v}=\sqrt{\frac{1}{2}\left[{\left(\sigma -0\right)}^{2}+{\left(0-0\right)}^{2}+{\left(0-\sigma \right)}^{2}\right]}$$

we get

(5) $$\sigma_{v}=\sqrt{\frac{1}{2}\left[\sigma^{2}+\sigma^{2}\right]}$$

It results in the following:

(6) $$\sigma_{v}=\sqrt{\frac{2\sigma^{2}}{2}}=\sigma$$

The von Mises stress simplifies to the following:

(7) $$\sigma_{v}=\sigma =\frac{F}{A}$$

Thus, in simple uniaxial tension, the von Mises stress is equal to the applied tensile stress, which is directly proportional to the force registered in the tensile test [27].

**Figure 13:**
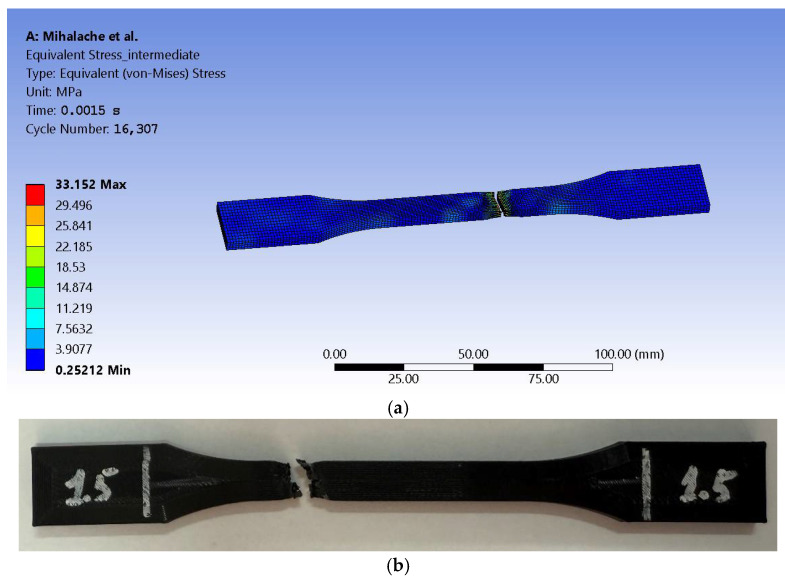
Graphical representation of a specimen: (**a**) simulation given equivalent (von Mises) stress distribution and (**b**) intermediate sample after the tensile test.

FEM shows a maximum value of 33.152 MPa (see Figure 13a) and averages for the mentioned value at 1.7474 MPa. Given that if we are to obtain direct proportionality, we would expect $\sigma_{v}=k\cdot F$, that would result in 1.02 for the k constant, which is consistent with the mathematical model given that this is an ideal simulation setup. Fracture shape and size are considered consistent between FEM and experimental tests in terms of visual conformance, as can be observed when compared to the image from Figure 13b.

By further exporting obtained results to the Steady-State Thermal module of Ansys, it was aimed to highlight thermal variation and distribution up to the necking stage. As it is known, fracture in a tensile test occurs after the material has exceeded its ultimate tensile strength (UTS). Out of the four stages it undergoes (i.e., elastic deformation, plastic deformation, necking, and fracture), FEM resulted in temperature values that were retrieved upon initiation of the third stage, whereas ultimate tensile strength is reached, the material reaches its maximum load capacity. That is because after necking, as the cross-section reduces, stress concentrates in the necked region.

**Figure 14:**
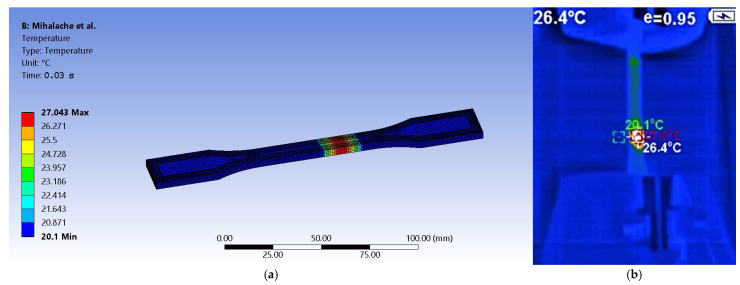
Graphical representation of thermal distribution: (**a**) according to FEM and (**b**) recorded live by thermal camera.

The 20.1 °C temperature value was maintained and assigned to the entire body. The results show a similar distribution as the one recorded by the thermal camera in the experimental test sessions. The simulation peak at 27.043 °C is close to the 27.3 °C recorded by the thermal capture equipment (see Figure 14). The difference between values is explained by quantifying the margin of error. First, it starts by computing the absolute difference between the presented values:

(8) Margin of Error = |27.3 − 27.043| = 0.257 °C

The resulting value gives the possibility to compute relative error as follows:

(9) $$\mathrm{Relative}\;\mathrm{Error}\;=\;\left(\frac{0.257}{27.3}\right)\;\times \;100\;\approx \;0.94\%$$

The computed deviation is less than 1%, which is typically found acceptable in thermal simulations. One has to take into account experimental uncertainties such as the precision of the sensor with ±2% error, ambient conditions, which may include uncontrolled airflow or heat losses, and even a slight spatial deviation when placing the probes from the modelled location. FEA modelling assumptions include material properties and possible mesh resolution.

The image in Figure 14b shows a screen capture from a video file and therefore is not at high resolution. Because the camera captures the evolution of temperature during tensile tests, it shows multiple values such as the ones for spot measuring at a specific moment in time as well as the last significant value recorded during the process.

The results show that as a material deforms, mechanical work is converted into heat due to plastic deformation and internal friction. The temperature rises due to the strain rate because if we achieve a faster deformation than more heat accumulation will occur as it is in the present case. Again, given the four stages of tensile tests that the specimen undergoes, one may conclude that, after yield strength, the temperature starts to increase, with the mention that higher strain rates lead to a more pronounced rise. Before fracture, localized temperature spikes in the necking region occur as thermal softening may accelerate failure by reducing local material strength. This phenomenon has been captured by FEM. As expected for the ABS, under load, the material shows a rather sharp temperature increase before fracture, which suggests significant plastic work absorption.

By corroborating simulation results with those obtained during experimental tests, one may observe the benefits of using thermal capture equipment in the form of the design of materials for high-temperature environments or high-strain-rate applications (e.g., aerospace and automotive), as well as being useful in fracture mechanics modelling, which may lead to improved failure predictions.

Authors recommend treating results with care since FEM uses an ideal model. Future analyses may take into account that some materials may exhibit slight cooling due to thermoelastic effects, which are interpreted as entropy changes. In addition, the material’s thermal conductivity may be assessed by means of thermal imagining, which would capture how quickly heat dissipates.

### 3.3. Scanning Electron Microscopy (SEM) Results

**Figure 15:**
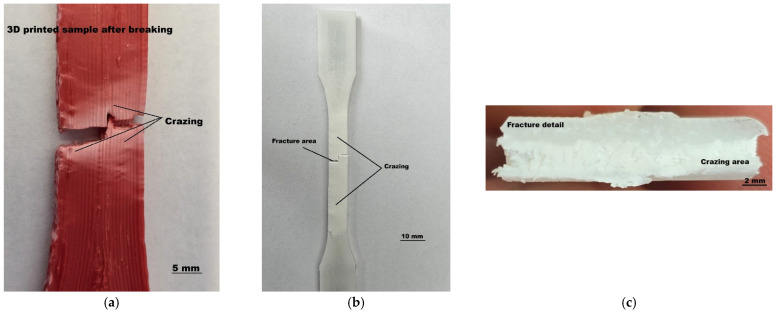
Snapshots of optical insights of various fractured samples: (**a**) ASA fractured sample; (**b**) ABS fractured sample; and (**c**) detail of the ABS fractured sample.

From a macroscopic point of view, all samples showed, in the middle area and especially on the edges, whitening of the material during the tensile test, until breaking, and afterward, it persisted (Figure 15).

When 3D-printed plastic samples turn white at the break area after a mechanical test, it is usually due to plastic deformation and microstructural changes in the material. The whitening is primarily caused by light scattering from internal damage (crazes, cracks, and voids) or changes in material structure caused by mechanical stress.

Under mechanical stress, microscopic voids and fibrils form in the polymer matrix—this is known as crazing. A more detailed explanation states that crazing is a stress-induced phenomenon in thermoplastics, where localized areas of plastic undergo microcrack formation. These microcracks are often filled with oriented polymer fibrils that can still carry load, making crazing a precursor to cracking, but not yet full structural failure, and they appear as white, hazy lines or zones, typically perpendicular to the direction of stress. Is common in amorphous thermoplastics like PLA, ABS, and PETG [28].

A different factor that can influence color change is plastic deformation through local yielding that leads to morphological changes in the polymer matrix. Commonly, crazing can manifest in 3D-printed samples due to residual stresses, material choice, environmental exposure, and processing conditions.

This whitening phenomenon originates at the center (highest stress concentration zone) and spreads toward the edges, especially being noticeable at surface layers where deformation is the most visible. It persists after fracture, which is an indication of permanent structural changes, as well as of ductile-to-brittle transition under certain raster angles or loading conditions [29].

First, we have analyzed SEM images (five for each sample) to determine the porosity and layer voids area of the samples using the ImageJ 1.54g (Wayne Rasband and contributors National Institutes of Health, Bethesda, MD, USA) image analysis software. For porosity, areas of 0.12 × 0.12 mm^2^ were analyzed and the results showed a higher porosity for samples 4.4, 5.5, and 12.3 (with values of 4.1%, 2.83%, and 2.09%, respectively), while samples 14.5, 22.2, and 23.2 had lower porosities below 1%: 0.9%, 0.3%, and 0.5%, respectively.

Regarding the analysis of layer voids and their distribution on the surface of the samples, five determinations were also performed; analyses on a surface of 1.56 mm^2^ and the following average values were obtained: for sample 4.4, 2.4%, for sample 5.5, 1%, sample 12.3, 6.9%, sample 14.5, 4.8%, sample 22.2, 1.1%, and sample 23.2, 3.1%.

**Figure 16:**
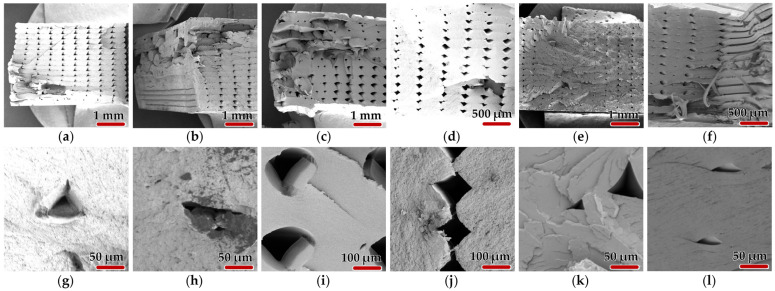
SEM related images of 3D-printed fractured samples at different magnification factors: (**a**) ABS 25x, sample 4.4; (**b**) ABS 25x, sample 5.5; (**c**) ASA 25x, sample 12.3; (**d**) ASA 50x, sample 14.5; (**e**) rPETG 25x, sample 22.2; (**f**) rPETG 50x, sample 23.2; (**g**) ABS 500x, sample 4.4; (**h**) ABS 500x, sample 5.5; (**i**) ASA 250x, sample 12.3; (**j**) ASA 250x, sample 14.5; (**k**) rPETG 500x, sample 22.2; and (**l**) rPETG 500x, sample 23.2.

Two SEM samples of each material were chosen to be presented. Graphical images were obtained at different magnification factors. It was chosen to present instances of both macro- and micro-scale with different topology and distribution of fractures so that for each material differences would be highlighted (see Figure 16).

It can be observed that each material is behaving differently. The results confirm the general knowledge about the three materials: the most ductile is recycled PETG, which typically has high elongation at break, often 20–30% or more; ASA comes next with a typical elongation at break around 10–20%; and ABS being the less ductile among the three, with elongation at break, usually around 5–15% [30]. In the case of ABS, one may observe that layer voids are in a straight line because of sudden fracture, and at the macro-level, the void keeps it shape as it elongates very little. In addition, on edges, one may observe the stacking of fibers, thus confirming the hypothesis of the proposed printing strategy. With ASA, layer voids are beginning to flatten as debonding occurs within the tested specimens (see Figure 16j). It makes fibrils break away and stack upon each other as voids begin to derail from straight lines. rPETG samples suffer the most, as full detachment is present between the layers as voids get flattened out towards the margins of the specimen. Some break away and form thin filiform threads (see Figure 16f).

The formation of flakes is also notable in the case of rPETG. It refers to thin, shard-like fragments that peel or splinter off from the fracture surface during or after the failure of a tensile specimen that are not smooth or ductile tears but rather brittle, lamellar separations or delaminated fragments from the fracture plane. Their formation may be caused by the fact that recycled PETG’s often consist of a mix of degraded polymer chains, contaminants, or additive residues. In this case, thermal evolution has a significant influence as molecular weight reduction from thermal history leads to temporal loss of ductility. Of course, one must take into account the fact that the proposed strategy of printing using rPETG is layer-by-layer, which leads to anisotropic strength that under stress delaminates, producing flake-like fracture patterns, especially along layer lines or flow lines (see Figure 16k). Repeated melting during recycling may also create heterogeneous crystallinity, which amplifies flaking.

## 4. Conclusions

When assessing thermal evolution, implications during tensile fracture refer to material intrinsic properties: ABS and ASA both have low *Cp* and *k*, which leads to more pronounced local temperature rise where it is presumed that heat remains localized, potentially softening the fracture zone. rPETG has a slightly higher *Cp* and *k*, therefore allowing for more thermal buffering and less localized heating but slower temperature spikes. One may conclude that the temperature rise during fracture is affected by how well the material absorbs (*Cp*) and dissipates (*k*, *α*) the heat generated during deformation. Tensile testing-wise, under smaller loads, ASA samples showed greater displacements. ABS samples need sustained loads, generally greater than ASA but do show cleaner, sharp fractures. The rPETG samples require the greatest loads among all but show displacements similar to ABS in values.

The present study focused on evaluating material behavior under quasi-static loading conditions using fixed strain rates. While this approach provides valuable insights into baseline mechanical performance, it is recognized that real-world applications often involve dynamic or impact loading with variable strain rates. Future work will aim to address these conditions to offer a more comprehensive understanding of the material response under practical service environments.

Scanning electron microscopy revealed that fracture surfaces of ASA material samples exhibit the smallest dispersion in fracture shape. Flakes are more pronounced in the case of rPETG given its modified form. Poor thermal bonding or oxidation may further weaken polymer chain entanglement as under an SEM microscope; fibrillar pull-out and voids between flakes may be observed. ABS samples are the cleanest ones in terms of fracture because of sustained loads and small displacements after tensile testing.

The combination of 3D-printed specimens, tailored process parameters, controlled testing conditions, and thermal imaging has proven effective in establishing a coherent thermal measurement system. The proposed approach demonstrated sufficient sensitivity in detecting variations in mechanical response in tensile testing. This unified methodology represents a viable alternative to conventional sensor-based systems, offering a non-intrusive, flexible, and cost-effective solution for real-time thermal evaluation in experimental and applied research contexts.

## Figures and Tables

**Table 1:** Taguchi L27 orthogonal design matrix.

Run Ord.	Material	Raster Height (mm)	Raster Width (mm)	Material Flow (%)	Test Speed (m/s) (mm/min)	Avg. Fracture Temp. (°C)	Avg. Load (N)	Avg. Displacement (mm)
1	ABS	0.15	0.5	100	50	37.66	37.663	45.165
2					100	50.16	50.163	60.165
3					200	75.16	75.163	90.165
4		0.25	0.6	105	50	38.96	38.963	46.705
5					100	51.46	51.463	61.705
6					200	76.46	76.463	91.705
7		0.35	0.7	110	50	40.26	40.263	48.245
8					100	52.76	52.763	63.245
9					200	77.76	77.763	93.245
10	ASA	0.15	0.6	110	50	40.19	40.188	48.195
11					100	52.69	52.688	63.195
12					200	77.69	77.688	93.195
13		0.25	0.7	100	50	37.74	37.738	45.235
14					100	50.24	50.238	60.235
15					200	75.24	75.238	90.235
16		0.35	0.5	105	50	38.96	38.963	46.685
17					100	51.46	51.463	61.685
18					200	76.46	76.463	91.685
19	rPETG	0.15	0.7	105	50	38.96	38.963	46.725
20					100	51.46	51.463	61.725
21					200	76.46	76.463	91.725
22		0.25	0.5	110	50	40.19	40.188	48.175
23					100	52.69	52.688	63.175
24					200	77.69	77.688	93.175
25		0.35	0.6	100	50	37.74	37.738	45.215
26					100	50.24	50.238	60.215
27					200	75.24	75.238	90.215

**Table 2:** Filament material comparisons.

Property	ABS	ASA	rPETG
Cp [J/g∙K]	1.3–1.6	1.4	1.0–1.20
k [W/m·K]	0.128–0.187	0.2	0.24–0.3
α [mm^2^/s]	0.11–0.17	0.13–0.18	0.12–0.18

**Table 3:** Analysis of variance of the sample’s temperature at fracture.

Source	DF	Seq SS	Contribution	Adj SS	Adj MS	F-Value	*p*-Value
Regression	8	121.530	64.50%	121.530	15.1912	4.09	0.006
Raster height (mm)	1	6.456	3.43%	6.456	6.4560	1.74	0.204
Raster width (mm)	1	0.605	0.32%	0.605	0.6050	0.16	0.691
Material flow (%)	1	5.423	2.88%	5.423	5.4230	1.46	0.243
Test speed (mm/min)	1	3.661	1.94%	3.661	3.6610	0.99	0.334
Material	2	66.641	35.37%	31.860	15.9300	4.29	0.030
Raster height × Raster width	1	17.613	9.35%	17.613	17.6131	4.74	0.043
Raster width × Material flow	1	21.131	11.22%	21.131	21.1313	5.69	0.028
Error	18	66.877	35.50%	66.877	3.7154		
Total	26	188.407	100.00%				

## Data Availability

Information supporting the reported results can be obtained upon request to the authors.

## Abbreviations

The following abbreviations are used in this manuscript:

ANOVA	analysis of variance
DOE	design of experiments
FEM	finite element method
SEM	scanning electron microscopy
DSC	differential scanning calorimetry
TGA	thermogravimetric analysis
PLA	polylactic acid
TPU	thermoplastic polyurethane
CF-PLA	carbon fiber-reinforced polylactic acid
PMMA	polymethyl methacrylate
Tg	glass transition temperature
GRA	grey relational analysis
TC	thermal conductive
TTS	time–temperature superposition
UTS	ultimate tensile strength
CPPS	cyber–physical production system
FEA	finite element analyses
FDM	fused deposition modelling
ABS	acrylonitrile butadiene styrene
ASA	acrylonitrile styrene acrylate
rPETG	recycled polyethylene terephthalate glycol-modified
PET	polyethylene terephthalate
